# Mechanochromic Detection for Soft Opto-Magnetic Actuators

**DOI:** 10.1021/acsami.1c11710

**Published:** 2021-10-01

**Authors:** Pau Güell-Grau, Pedro Escudero, Filippos Giannis Perdikos, José Francisco López-Barbera, Carlos Pascual-Izarra, Rosa Villa, Josep Nogués, Borja Sepúlveda, Mar Alvarez

**Affiliations:** † Instituto de Microelectrónica de Barcelona (IMB-CNM, CSIC), Campus UAB, Bellaterra, 08193 Barcelona, Spain; ‡ Departament de Física, Universitat Autònoma de Barcelona, 08193 Bellaterra, Spain; § ALBA Synchrotron, 08290 Cerdanyola del Valles, Spain; ∥ Networking Research Centre on Bioengineering, Biomaterials and Nanomedicine (CIBER-BBN), 28029 Madrid, Spain; ⊥ Catalan Institute of Nanoscience and Nanotechnology (ICN2), CSIC and BIST, Campus UAB, Bellaterra, 08193 Barcelona, Spain; # ICREA, Pg. Lluís Companys 23, 08010 Barcelona, Spain

**Keywords:** mechanochromic, structural coloration, opto-magnetic, soft actuator, smart sensing

## Abstract

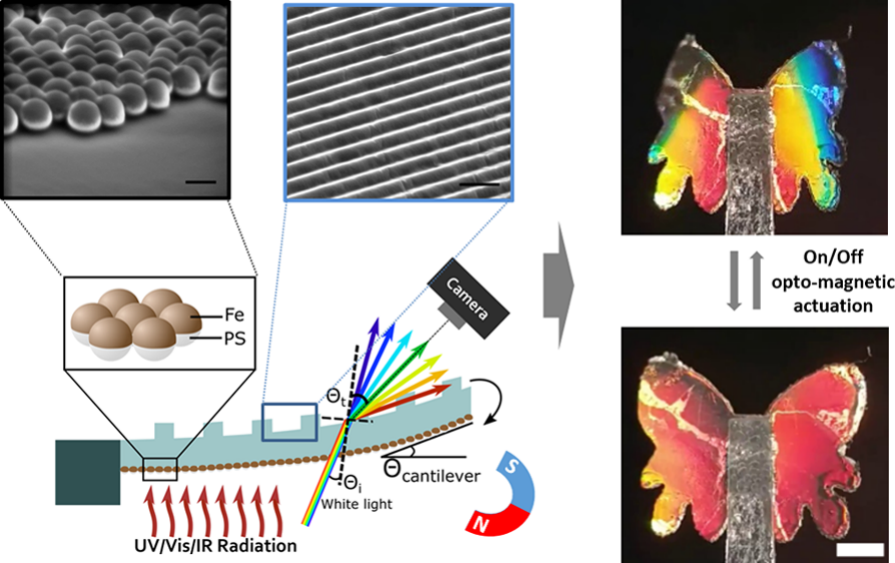

New multi-stimuli responsive materials are required in smart systems applications to overcome current limitations in remote actuation and to achieve versatile operation in inaccessible environments. The incorporation of detection mechanisms to quantify in real time the response to external stimuli is crucial for the development of automated systems. Here, we present the first wireless opto-magnetic actuator with mechanochromic response. The device, based on a nanostructured-iron (Fe) layer transferred onto suspended elastomer structures with a periodically corrugated backside, can be actuated both optically (in a broadband spectral range) and magnetically. The combined opto-magnetic stimulus can accurately modulate the mechanical response (strength and direction) of the device. The structural coloration generated at the corrugated back surface enables to easily map and quantify, in 2D, the mechanical deflections by analyzing in real time the hue changes of images taken using a conventional RGB smartphone camera, with a precision of 0.05°. We demonstrate the independent and synergetic optical and magnetic actuation and detection with a detection limit of 1.8 mW·cm^–2^ and 0.34 mT, respectively. The simple operation, versatility, and cost-effectiveness of the wireless multiactuated device with highly sensitive mechanochromic mapping paves the way to a new generation of wirelessly controlled smart systems.

## Introduction

Soft actuators and stimuli-responsive materials are attracting increasing attention in soft robotics, artificial muscles, wearables, and biomimetic devices^1−4^ due to their ability to deform and adapt to different environments with high resiliency. In these systems, the capacity of remote actuation by wireless stimuli, such as light or magnetic fields, can prevent the need for wiring and electrical contacts, thereby greatly reducing the complexity of the actuators and enabling their operation in hazardous environments or not easily accessible spaces.^5,6^ Both optical and magnetic actuators have been extensively studied, however, each of them faces its own challenges and limitations.^7^ The optical actuation of soft robots^8−10^ is generally based on photo-thermo-mechanical effects, in which the absorbed light is converted into heat to generate the mechanical actuation. Hence, many efforts are currently devoted to the development of efficient, broadband light absorbers that can be implemented into soft mechanical structures.^11−13^ However, light-triggered actuation is limited by the slow cooling processes after actuation and to environments that are transparent to the actuating light. Soft magnetic actuators provide an interesting alternative due to their ability to perform robust and programmable shape changes^14^ and precise manipulation of the structures^15^ over long distances or through opaque media, which are very attractive for biomedical applications.^16,17^ Therefore, the integration of multi-stimuli responsive materials presents exciting possibilities for the new generation of soft robotics. For example, light and magnetic field actuation can be combined to double the response strength, generate independent actions (such as bending and rotation),^18,19^ or modulate one actuation using the other one as a stimulus.^20,21^ In addition, combining various stimuli overcomes the specific limitations in the applicability of each stimulus, hence, expanding the overall application range. Composite films comprising magnetic iron (Fe) particles are especially interesting for achieving such combined light and magnetic actuation,^18,19,21^ due to their capability to absorb light (photothermal response) and their strong ferromagnetic response; however, the controlled photothermal and magnetic actuation of polymer composites for soft robotics has been rarely exploited.^21^

In addition to an efficient wireless actuation, detection of the actuation strength is key to enable automatic and precise movement control based on real-time feedback signals.^22,23^ However, the integration of detection mechanisms into soft actuators remains a challenge, especially for small structures (i.e., less than a centimeter).^24^ Current actuation control involves either detecting the stimuli intensity at the actuated structure or directly quantifying its mechanical response. The direct detection of the stimuli, which implies the integration of external sensors in small, movable structures^25−27^ is technologically complex. Conversely, current methods to quantify the mechanical response involve either incorporating complex wired electrical contacts and piezoelectric materials,^3,22,28−30^ using external bulky laser positioning systems with limited multiplexing capabilities, or analyzing the shape changes *via* complicated and inefficient imaging analysis, especially for small structures. To solve these technological issues, detection based on structural coloration presents several advantages, such as wireless and real-time sensing of the actuation strength.^31,32^ However, the colorimetric detection in wirelessly controlled actuators requires the use of soft materials with sufficient light reflectance or transmittance, and the real-time readout and analysis should be based on image processing rather than on spectrometry to successfully integrate and simplify the detection and to enable fast 2D quantification mapping.^33−35^

Here, we integrate structural coloration into a dual opto-magnetic soft actuator to achieve highly sensitive mechanochromic detection of the wireless actuation with light and/or magnetic fields. We demonstrate an efficient dual opto-magnetic actuation by exploiting the ultrabroadband optical absorption and the strong magnetic response of a novel nanostructured-Fe/polydimethylsiloxane (PDMS) metamaterial.^13^ The nanostructured-Fe plays a critical role in both the optical and magnetic actuation, by enhancing the thermoplasmonic effect in a broadband spectral range and producing an in-plane (IP) ferromagnetic vortex configuration showing high linear magnetic susceptibility and low saturation field,^36,37^ thereby improving the actuation efficiency and control with respect to Fe thin films or Fe nanoparticles. The structural coloration is based on a periodically corrugated backside on the PDMS layer acting as a bendable diffraction grating. The color changes of the actuators are processed in real time by a 2D imaging algorithm that quantifies the hue (H) value variations in the selected regions of interest (ROI) of the images taken using a conventional RGB camera or a smartphone camera. This approach would enable the “remote” operation of the system, both actuation and detection, by adjusting the size of the sensor and the optics of the camera to achieve a proper spatial resolution even for long working distances.

## Results and Discussion

**Figure 1:**
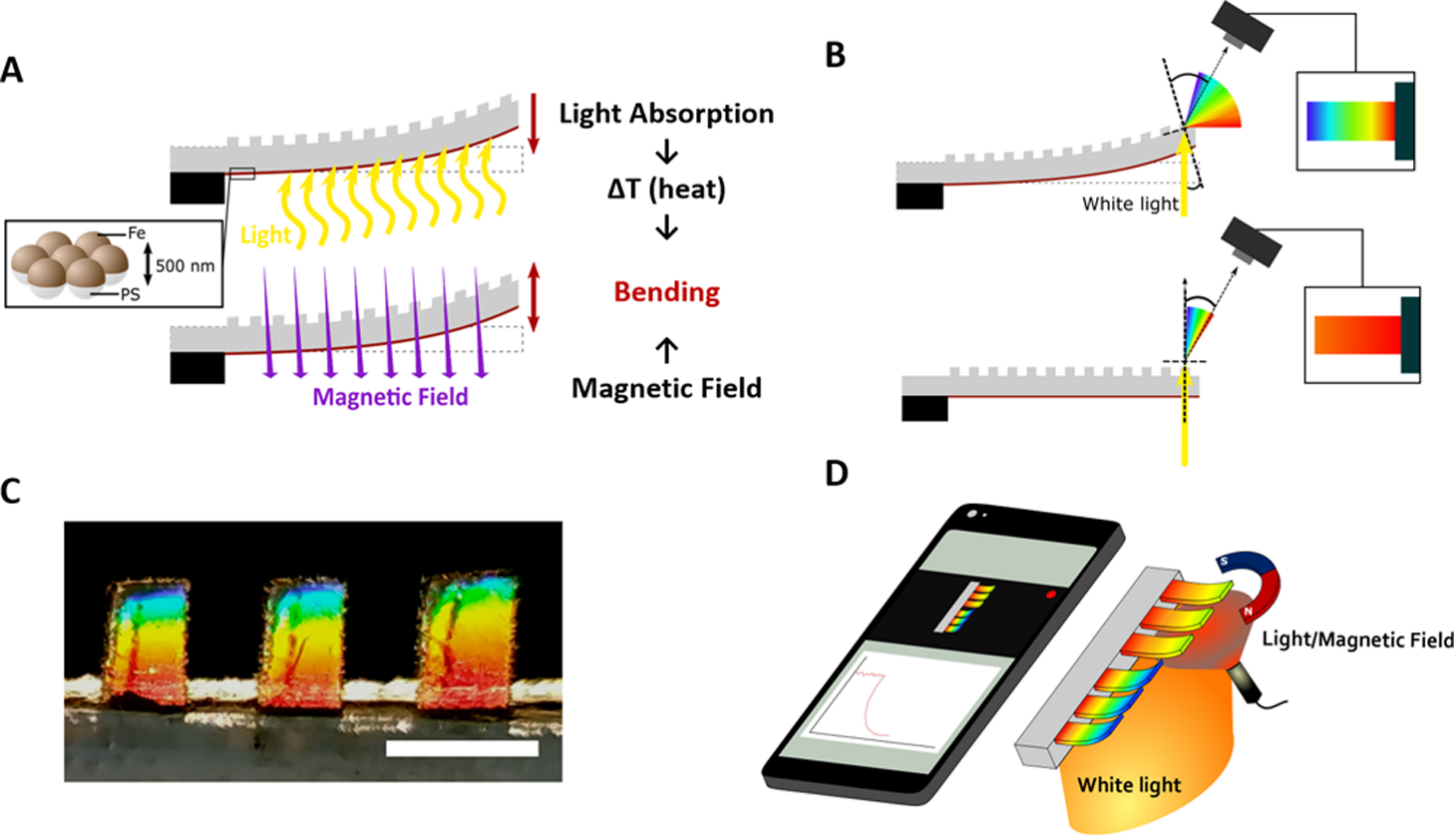
(A) Schematic of a cantilever-shaped soft opto-magnetic actuator with integrated mechanochromic detection, composed of a nanostructured-Fe film mechanically linked to a periodically corrugated thin PDMS, and schematic of the light and magnetic actuation induced bending. The images depict the two actuation modes, light (top) and magnetic fields (bottom). (B) Schematic of the structural coloration generated by the white light diffraction into the grating surface. (C) Photograph of the fabricated cantilever array under diffracted white light in transmission mode taken with a smartphone (scale bar = 1 mm). (D) Cartoon of the complete setup.

The concept of combining photothermal heating, magnetic actuation, and mechanochromic sensing is demonstrated in a cantilever-shaped actuator. The soft opto-magnetic actuator with mechanochromic detection is composed of interconnected semi-shell Fe nanostructures (500 nm) with an average thickness of 80 nm, referred as nanostructured-Fe film, mechanically coupled to a suspended 50 μm thick PDMS film whose back surface is periodically corrugated (i.e., grating) (Figure 1A). Due to its highly damped plasmonic response,^13^ the nanostructured-Fe absorbs light, which effectively heats the system and induces a bending response (Figure 1A). In addition, the nanostructured-Fe exhibits ferromagnetic properties that can be used to bend the actuator in opposite directions depending on the magnet orientation (Figure 1A). Note that Fe has a very high Curie temperature, which provides a robust light-independent magnetic actuation and guarantees the independent device actuation by light and magnetism. On the other side of the structure, an incident white light is diffracted by the grating surface (Figure 1B), producing a color gradient along the cantilever length, depending on the cantilever curvature (Figure 1C). Any change in the cantilever bending is instantaneously converted into a shift in the diffracted color (i.e., mechanochromism) which enables the color change mapping and the actuation/bending quantification by using, for example, a smartphone camera (Figure 1D).

**Figure 2:**
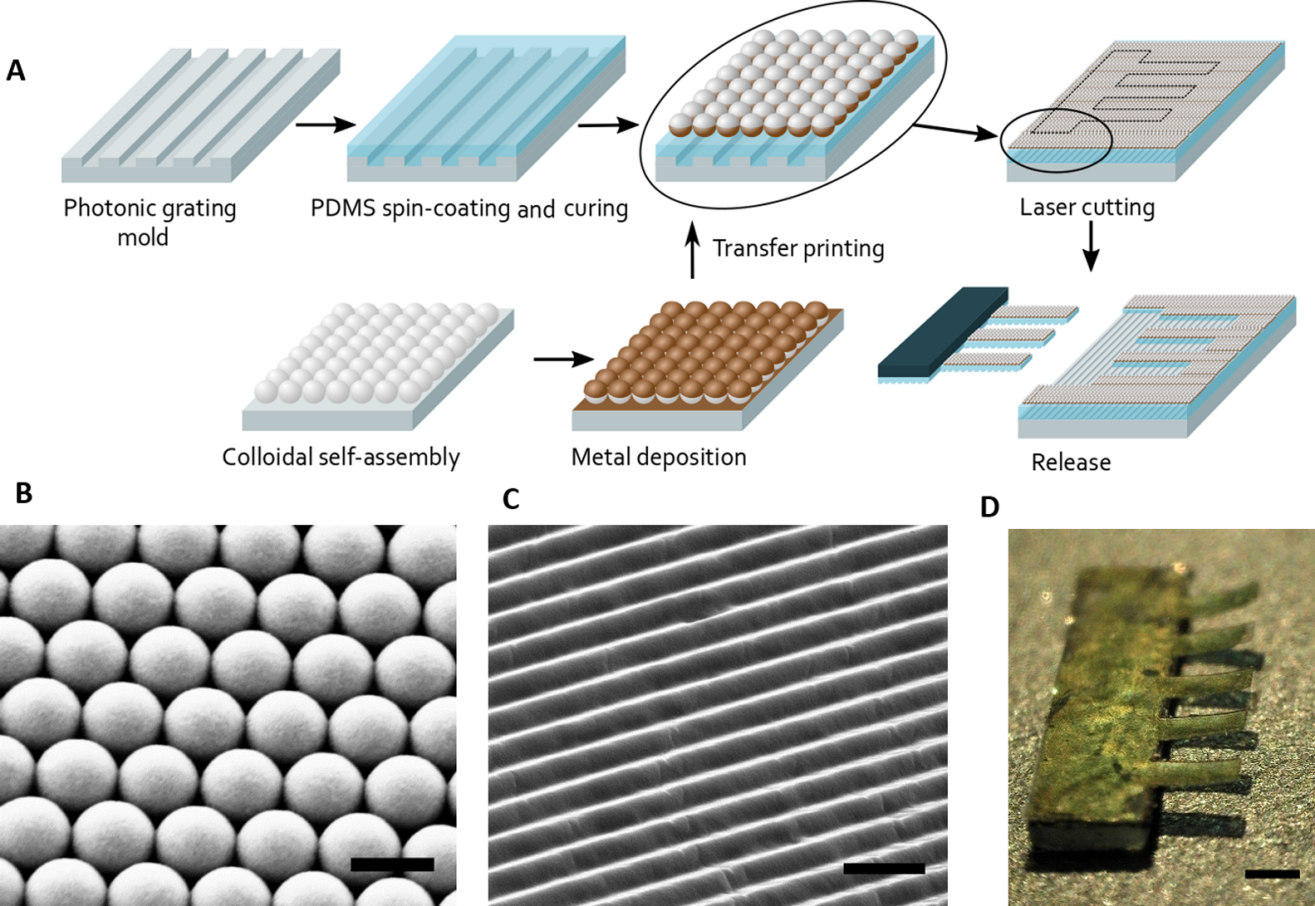
(A) Schematic of the fabrication process. (B) SEM image of the nanostructured-Fe film (scale bar = 500 nm). (C) SEM image of the PDMS photonic grating (scale bar = 2 μm). (D) Photograph of the fabricated cantilever array under ambient light (scale bar = 1 mm).

To produce the soft mechanochromic opto-magnetic actuators, a nanostructured-Fe layer obtained by colloidal lithography is transferred onto the flat surface of a PDMS layer with an opposite periodically corrugated surface (Figure 2A). The nanostructured-Fe layer fabrication starts by forming a monolayer of self-assembled hexagonal close-packed polystyrene beads (diameter 500 nm) on a Si substrate, followed by the deposition of 80 nm of Fe on them by electron beam evaporation^38,39^ (Figure 2B). In parallel, the 50-μm-thick bendable photonic grating is obtained by spin-coating PDMS on a periodically corrugated silicone mold casted from a commercial diffraction grating (pitch 1600 nm) (Figure 2C). The thickness homogeneity of the cantilevers was controlled by profilometry. Next, the nanostructured-Fe layer is mechanically coupled to the flat surface of the PDMS film by transfer printing, that is, by mechanically pressing the PDMS against the substrate with the Fe nanostructures. Finally, the size and shape of the suspended structures are defined by laser writing and released from the grating mold. For the mechanochromic and opto-magnetic analysis, the freestanding structures have a rectangular shape with a length and width of 1500 and 600 μm, respectively (Figure 2D). The size was selected to have a good compromise between the rigidity of the structure and its visualization using colorimetric imaging (by using basic optics). The released freestanding structures present an initial curvature due to the mechanical stress generated in the transfer of the nanostructured metal film and in the final release. Nevertheless, although this last manual process yields slight initial curvature variations between different cantilevers, their opto-magnetic response is analogous.

The periodically patterned PDMS surface provides the mechanochromic response by diffracting the incident white light, thus giving the structural coloration to the freestanding actuator, and changing its structural diffracted color in response to the experienced optical/magnetic stimuli. The light diffraction is described by Bragg’s law: *m*λ = *d*(sin(θt) – sin(θi)), where *m* is the diffraction order, λ is the wavelength, d is the grating pitch, and θ*t* and θ*i* are the transmitted and incident angles of the light relative to the surface normal. For a constant grating pitch and diffraction order, the observed wavelength (i.e., color) at a specific observation angle depends only on the light incident angle with respect to the cantilever curvature. Therefore, any angular deflection of the structure is instantaneously converted into a structural color change. Note that the fabricated freestanding structures present an initial curvature toward the PDMS grating caused by the residual mechanical stress between the metal and polymer layers, which is responsible for the color gradient observed under white light diffraction before any external actuation (Figure 1C). The initial upward bending is exploited to achieve a higher flexibility on the cantilever actuation, allowing displacements from positive to negative bending with respect to the flat position. Note that following Bragg’s equation, changes in the pitch of the grating may also produce a change in the diffracted color due to an inhomogeneous periodicity along the cantilever or changes in the periodicity due to the experienced strain during the bending. However, in a bent cantilever system, the maximum strain is produced near the clamped end which would produce a color gradient from red (at the cantilever free end) to blue (at the cantilever clamped end), which is opposite to the observed color gradient. Also note that the pitch of the patterned grating is very homogeneous (Figure 2C). In view of this, we consider that the largest contribution to the color changes arise from the variation of the angle.

The mechanical status of the freestanding structures is therefore monitored in real time with an RGB camera by using an open-source algorithm (Figure S1A) that obtains the color shade, that is, the H value (from the hue–saturation–lightness color space)^40^ in the pixels selected by the defined ROI. To convert from light wavelength (color) to its corresponding H value in hue degrees (H-deg), it is usually assumed that 0 H-deg corresponds to 650 nm (red) and 270 H-deg to 400 nm (blue)^41^ (Figure S1B). Remarkably, this colorimetric mechanochromic detection approach based on 2D H maps provides a high spatial resolution, which is not feasible using colorimetric systems based on spectroscopic measurements.^42−44^ The high spatial resolution enables direct visualization of strain gradients and the simultaneous analysis of individual freestanding structures for multiplexed actuation/detection systems. In our case, in which the camera is located at approximately 3 cm from the structures, the spatial resolution is approximately 7 μm, which could be substantially enhanced using high numerical aperture objectives with higher magnification. Moreover, the image analysis software allows simultaneous monitoring of the H value changes in several different ROIs, which is essential for multiplexing applications.

This mechanochromic approach is very effective to detect the mechanical deflections in air, where the PDMS refractive index enables the efficient diffraction of the incident white light. In principle, the same approach could be used in liquids;^45^ however, a flexible substrate with higher refractive index should be used.

The selection of the nanostructured-Fe on the PDMS as active material is motivated by the possibility to generate single and dual actuation based on their very efficient optical and magnetic actuations. The optical actuation is based on the photo-thermo-mechanical effects of a bimorph actuator, where the absorbed light by either the nanostructured-Fe or the PDMS layers produces a temperature-induced mechanical surface stress that bends the structure.^8^ The mechanical deflection is proportional to the temperature changes (Δ*T*) and the difference in thermal expansion coefficients (α) of the materials (Supporting Information). Therefore, the high photothermal conversion efficiency combined with the large difference in the thermal expansion coefficient between the Fe and PDMS layers (i.e., α_*Fe*_ = 1.1 × 10^–5^ K^–1^ and α_PDMS_ = 3.1 × 10^–4^ K^–1^) are crucial to maximize the photo-thermo-mechanical response of the actuators. The ultrabroadband absorption in the whole IR range enables light actuation outside the visible spectrum, consequently, avoiding any crosstalk with the colorimetric detection system in the visible range. As it was analyzed in detail in ref (13), the ultrabroadband absorption range is achieved by the synergistic absorption of the nanostructured-Fe layer in the visible and near-infrared (NIR) and the PDMS layer in the mid/long wave infrared (MWIR–LWIR) (Figure S2A), which yields an average absorbance of 84% in the 0.4–18 μm range (minimum 75% @ 1250 nm–maximum 94% @ 530 nm). Consequently, any light source in this range could be equally used for the opto-mechanical actuation. It is worth emphasizing that the highly damped plasmons of the Fe nanostructures produce very broad resonances, further broadened by the connection between structures. As a result, an absorption peak is not observed and an ultrabroadband absorption is generated. The observed peaks and dips at short wavelengths are due to photonic crystal effects generated by the hexagonal close-packed structure of the nanostructured-Fe layer. However, the optical response in the NIR and IR, which are the spectral regions used for the opto-mechanical actuation, is nearly flat. The size of the Fe nanostructures, which is given by the diameter of the polystyrene spheres, has a weak influence on the shape of the absorption spectra (Figure S2B), particularly in the NIR and MWIR–LWIR ranges that show a rather flat response. Thus, the performance of the device should be largely unaffected by the size of the Fe nanostructures. However, reduction of the thickness of the deposited Fe layer decreases the overall absorption in the visible and NIR ranges (Figure S2B), due to the enhanced light transmission in that range, which would weaken the response. In contrast, the polystyrene spheres generate a minimal photothermal contribution, as they are transparent in the NIR and their absorption is negligible compared to that of the PDMS layer in the MWIR–LWIR ranges.

**Figure 3:**
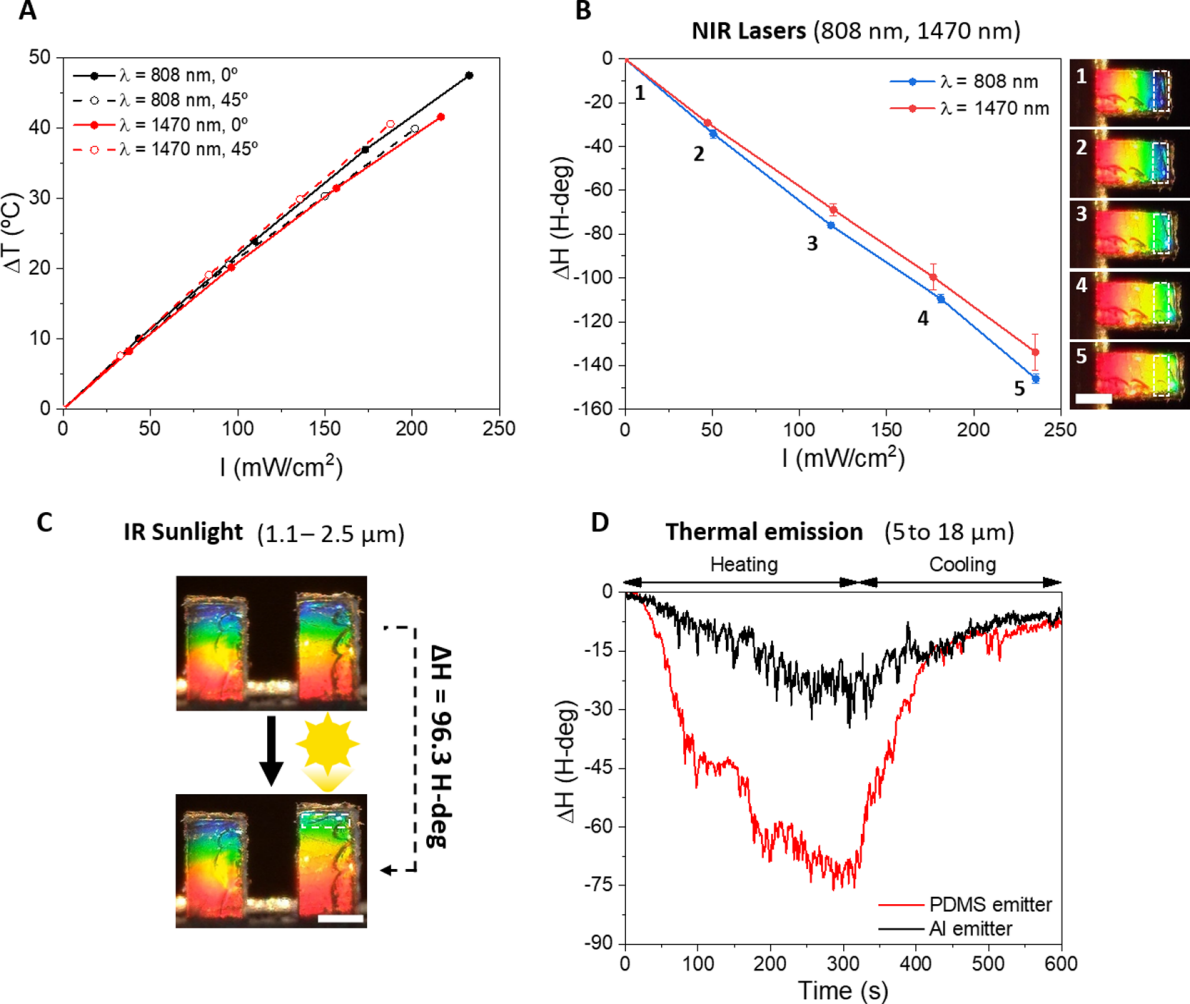
(A) Photothermal characterization of the nanostructured-Fe/PDMS for two different wavelengths (808 and 1470 nm) and two different angles of incidence (0 and 45°). (B) Colorimetric response of the mechanochromic actuator to NIR light (λ = 808 and 1470 nm) with representative pictures showing the large color changes. Scale bar = 0.5 mm. The rectangle marks the ROI used for the analysis. (C) Color shift generated by the sunlight filtered NIR–MWIR radiation focused on the right-side cantilever. Scale bar = 0.5 mm. (D) Color variation curve of the Fe/PDMS metamaterial actuated by the thermal IR emission radiated from PDMS or Al pieces (area 4 cm^2^) heated at 110 °C.

Importantly, the absorbed light is efficiently converted into thermal energy with minimal wavelength and incident angle dependence (due to its nanostructured morphology), as shown in Figure 3A. Namely, a linear dependence of the induced temperature increase with the light irradiance is observed, with similar heating rates at 808 and 1470 nm (0.21 and 0.20 K cm^2^ mW^–1^, respectively), in accordance with the nearly flat broadband absorption. Moreover, the heating rate is virtually independent of the light incident angle (0 or 45°) for both wavelengths.

The optomechanical actuation of the structures is characterized by exploiting the developed mechanochromic analysis (Figure 1). Images of the cantilever colored surface and the cantilever profile were taken simultaneously to correlate the color change and the cantilever bending when exposed to light radiation (Figure S3A). Moreover, to evaluate the homogeneity of the heating the temperature of the heated surface at a fixed power radiation was also imaged using a thermal camera (Figure S3B). Figure 3B shows the color change (i.e., the change in *H*, Δ*H*) at the cantilever free end (which is the region showing the largest deflection), as a function of the light intensity (*I*) for the two NIR stimulation wavelengths (808 and 1470 nm). The light absorption causes a H reduction, which implies a decrease in the cantilever angle. Namely, the cantilever flattens its initial curvature upon the NIR light actuation. The color shift in H-deg provides a direct quantification of the mechanical response. The opto-magnetic mechanochromic structure exhibits very notable color changes upon light irradiation, showing similar sensitivities (*S* = d*H*/d*I*) at 808 and 1470 nm (i.e., *S*_Fe-808_ = −0.62 H-deg·cm^2^·mW^–1^, *S*_Fe-1470_ = −0.57 H-deg·cm^2^·mW^–1^), as expected from the photothermal characterization. Considering that the experimental noise in the detection of the hue changes is 0.35 H-deg, the minimum detectable NIR light irradiance is 1.8 mW·cm^–2^ (see Experimental Section). It is important to highlight that, even though the nanostructured-Fe is able to absorb a significant percentage of the white light used for the mechanochromic detection, the incident irradiance (ca. 1 mW·cm^–2^) is sufficiently low to avoid any observable effect on the actuation. Furthermore, by correlating the light-induced angular deflection (measured from the cantilever profile by image analysis) and the hue change (i.e., 19.2 ± 0.5 H-deg/° according to Figure S3A), the minimum detectable deflection is as low as 0.05° (see Experimental Section). The opto-mechanical actuation is also correlated with the photothermal analysis to determine the relationship between the color shift and the induced temperature (Figure S3B). There is a nearly linear correlation between the color change and the temperature, which is identical for both laser sources at 808 and 1470 nm. This analysis enables establishing a sensitivity of −3.0 H-deg·K^–1^ and a detection limit of 0.35 K, which can also be the base for innovative low-cost wireless temperature detectors.

Due to the ultrabroadband light absorption of the nanostructured-Fe/PDMS structure, the mechanical actuation can be also performed by natural light sources, such as the sunlight and even the weak infrared thermal emission from a heated body. The actuation strength of the NIR–MWIR spectral part of the sunlight is quantified by filtering out the visible and UV sunlight with a silicon lens. The use of the silicon lens has two purposes: (i) to block all wavelengths below 1.1 μm and (ii) to focus the remaining low intensity radiation (from 1.1 to 2.5 μm) into a single mechanochromic structure. The focused radiation yields a visually observable color shift (96.3 H-deg) in comparison to the non-irradiated neighboring structures (Figure 3C). Considering the previous angle and temperature calibration curves, an angular deflection of 5.2° and a 32.5 K temperature increase is produced in the irradiated suspended structure by the absorbed NIR–MWIR sunlight (while the other structure remains unaltered).

Additionally, the weak thermal emission in the MWIR–LWIR range from two emitters with very different emissivity (ε), PDMS (ε = 0.86) and aluminum (Al) (ε = 0.04),^46^ are used to demonstrate the capacity of actuation by a broadband thermal emitter and to rule out any major effect of the air temperature changes in the response of the cantilevers. The emitters are heated up to 110 °C, which corresponds to a peak wavelength of 7.5 μm, according to the blackbody radiation spectrum. Figure 3D shows the large differences in the real-time color shifts generated during the heating and cooling of both emitters (ca. 72 and 23 H-deg for the PDMS and Al emitters, respectively). Since the Al emissivity is very close to 0, and the measurements are not performed in vacuum, it can be assumed that the observed color shift in this case arises only from the heated air, which should be similar for both emitters. Consequently, the contribution of the IR thermal emission from the PDMS emitter can be estimated by subtracting the Al signal, thus achieving a total MWIR–LWIR actuation signal of 49 H-deg.

**Figure 4:**
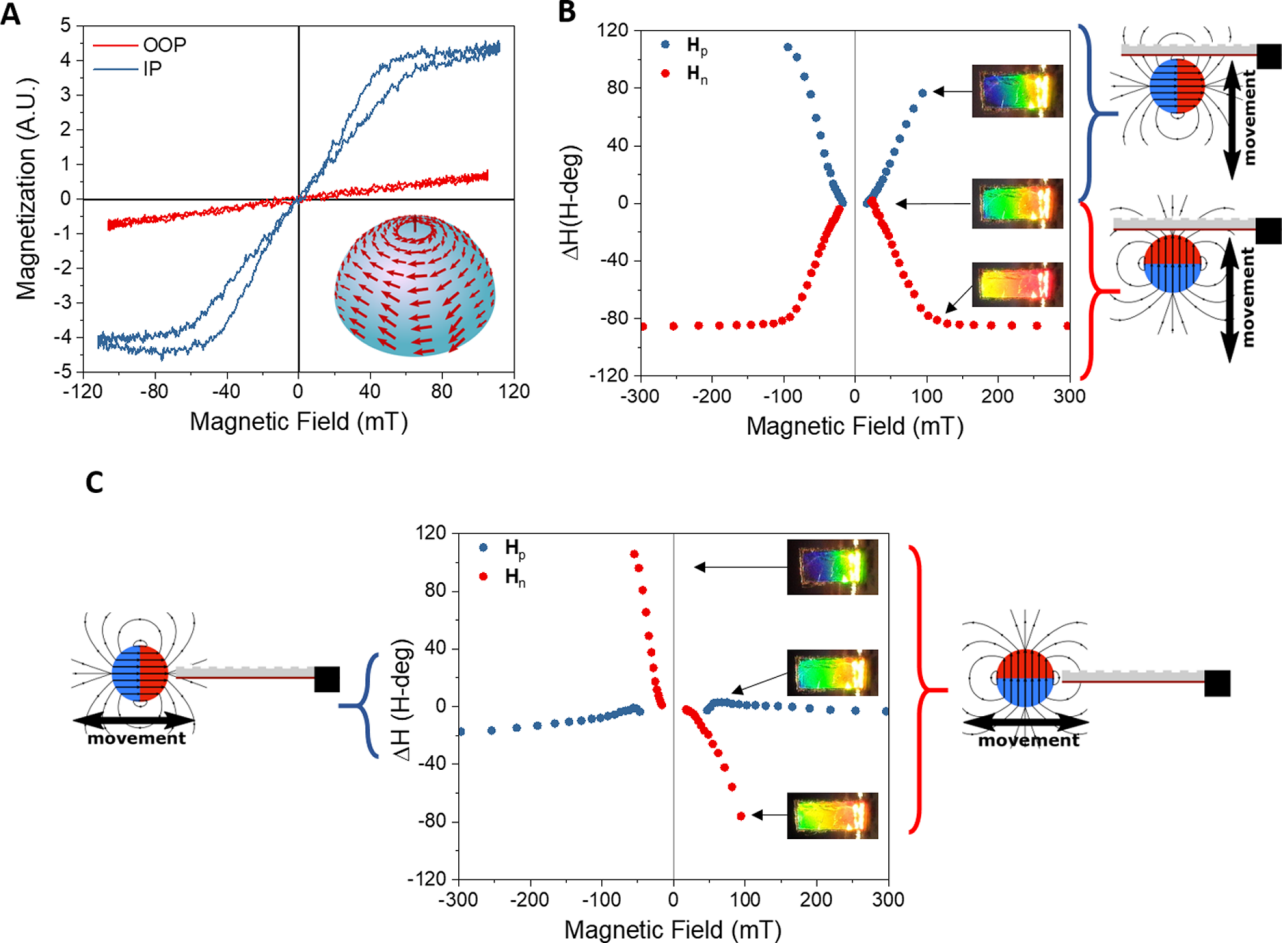
(A) Characterization of the IP and OOP magnetic response of the nanostructured-Fe/PDMS to an external magnetic field by magneto-optical Kerr effect (MOKE). Shown in the inset is the schematic representation of the magnetic moments at zero field, that is, a vortex configuration. (B,C) Colorimetric detection of the magnetic actuation of a magnet moving in the direction (B) normal to the cantilever plane and (C) parallel to the cantilever, as shown schematically by the different cartoons. The images in panels (B,C) correspond to the colorimetric responses of the cantilevers to diverse field conditions.

In addition to the optical actuation, the magnetic character of the nanostructured-Fe layer enables the actuation using external magnetic fields. Interestingly, the semi-shell structure of the Fe layer confers it a magnetic response rather different from the soft ferromagnetic response expected for a polycrystalline flat Fe layer (i.e., a square IP loop with a small coercivity). The IP hysteresis loop evidences that the nanostructured-Fe exhibits IP ferromagnetic vortex configuration,^36,37^ where the magnetization of each Fe semi-shell curls around the circular structure (see schematic representation in Figure 4A), leading to a linear and high susceptibility at low fields with nearly zero remanence and a negligible coercivity (Figure 4A). The out-of-plane (OOP) hysteresis loop (Figure 4A) corresponds to a hard-axis behavior, indicating that the magnetization is in the film plane at low fields. The size of the nanostructure and the Fe thickness can modify this magnetic response. For structures with a smaller diameter, the vortex-like magnetization reversal loops are more tilted, thereby showing a higher low field magnetic susceptibility. Moreover, for very thin Fe films (below 30 nm thickness) the magnetic response can change from a vortex to a single-domain behavior showing substantial magnetic remanence.^39^

The linear response at low fields with a high magnetic susceptibility of the magnetic vortex-like behavior of the structures offers versatile and efficient actuation capabilities based on the combination of magnetic torques and magnetophoretic forces (induced by field gradients), depending on the configuration of the applied external field, as explained in the Supporting Information. The magnetic actuation is characterized by analyzing the mechanochromic response of the freestanding structure to the magnetic field generated by a spherical permanent magnet (FeNdB, 12 mm diameter; see schematic representation of the field lines in Figure 4B,C). Note that the field lines of a uniformly magnetized sphere are equivalent to those of a magnetic dipole.^47^ The actuating magnetic field strength and direction is controlled by (i) moving the magnet with respect to the structure either parallel or perpendicular to the structural plane and (ii) changing the orientation of the magnet poles with respect to the freestanding structures (see Figure 4B,C). The induced magnetic actuation arises mainly from a complex combination of magnetic torques and magnetophoretic forces by the field gradient (Figure S5 and Supporting Information),^48,49^ since the magnetostrictive contribution in Fe is expected to be much weaker. The relative contribution of each mechanism (torque and magnetophoretic) depends on the orientation of the magnetic field with respect to the bent nanostructured-Fe layer. Consequently, the response of the cantilever is different depending on the position and orientation of the magnet with respect to the cantilever (see Figures 4B,C and Figure S5 and Supporting Information), and a simple analytic equation to predict the magnetic response cannot be easily established. As can be seen in Figures 4 and Figure S5, the freestanding structures are very sensitive to the applied magnetic field except for one of the configurations. For example, when the magnet is approached from under the cantilever (Figure 4B) the magnetic sensitivity is −1.1 and 1.1 H-deg/mT for the two orientations of the magnet, respectively. On the other hand, when the magnet is approached along the structures (Figure 4C), the sensitivity is even larger, reaching −3.1 H-deg/mT and a detection limit of 0.34 mT when the direction of the poles of the magnet is perpendicular to the plane of the cantilever (see Supporting Information). In contrast, the cantilever hardly moves when the magnet is approached along the structure with the direction of the poles parallel to the cantilever plane, since both the applied field and its gradient are mainly parallel to the nanostructured-Fe layer. Thus, both magnetophoretic and torque effects are expected to be small (see the Supporting Information). Finally, it is worth emphasizing that this strong and versatile magnetic actuation can be achieved with only a tiny fraction of ferromagnetic material in the structure, as the nanostructured-Fe layer constitutes barely 0.5% of the total mass of the actuator, and applying low magnetic fields.

**Figure 5:**
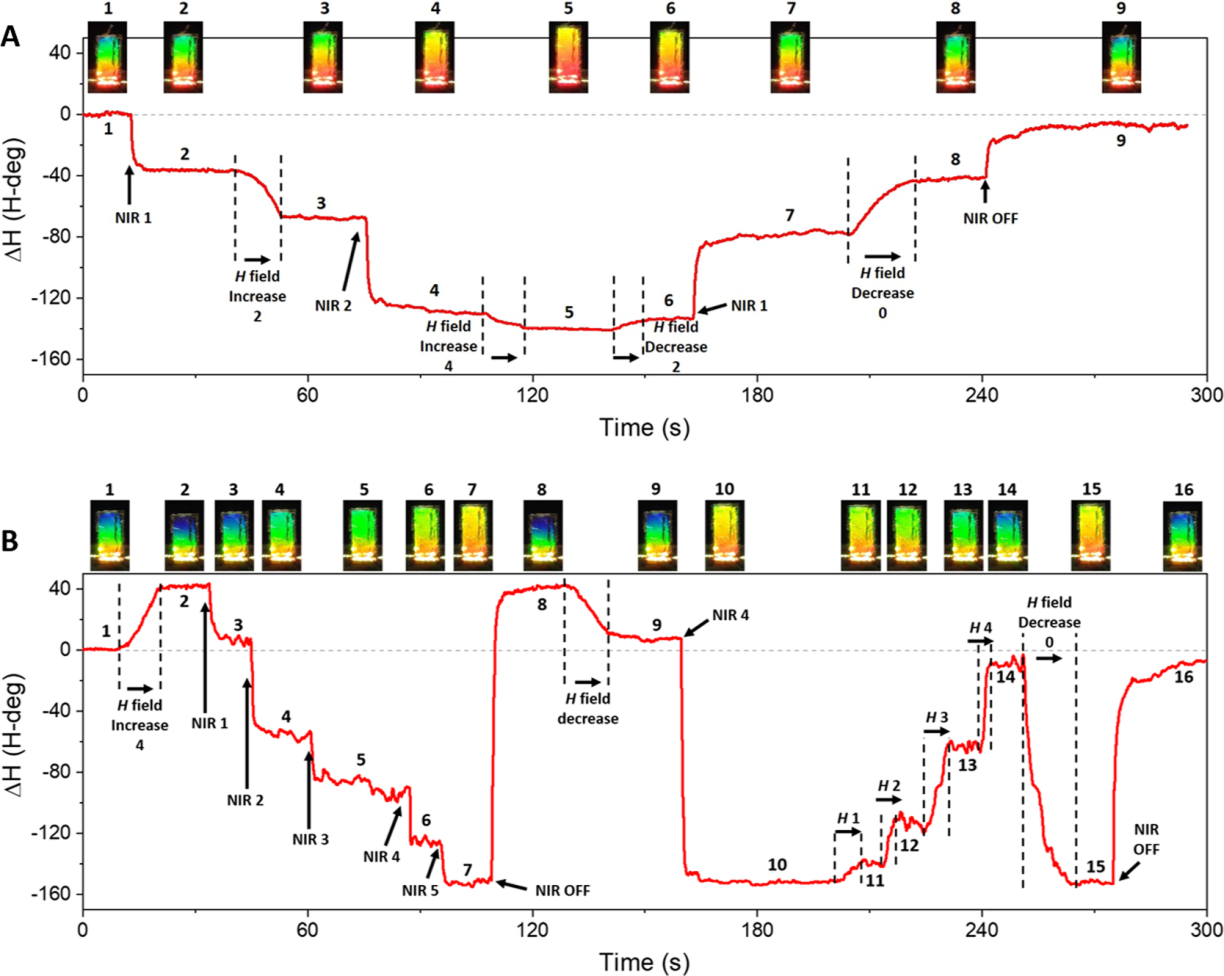
Panels (A,B) are two different combinations of light and magnetic field, adding their actuation (A) and counteracting each other (B). The magnet is oriented with respect to the structure as in Figure 4C. The diverse pictures show representative images of the colorimetric response of the structure at each stage. Note that laser intensities “NIR 1, 2, 3, 4, and 5” correspond to 75, 150, 225, 300, and 375 mW/cm^2^, respectively. The magnetic field changes “*B* 1, 2, 3, and 4” correspond to 35, 43, 55, and 71 mT, respectively.

To demonstrate the full versatility of the structures, we combine the optical and the magnetic actuation (Figure 5). Notably, dual opto-magnetic soft actuators, where the mechanical structure can be actuated by both light and magnetic fields, are very scarce.^21,50^ Although other actuators using light and magnetic fields can be found in the literature, the role of the magnetic field is merely to transport or rotate the structures, rather than for mechanical actuation.^18,19,51,52^ Since the magnetic actuation can drive the structure in both directions and the actuation mechanisms do not interfere between each other, we explore two different configurations by positioning the magnet to either (i) sum the optical and magnetic forces (Figure 5A, Video S1) or (ii) to counteract the optical actuation by magnetic forces and vice versa (Figure 5B, Video S2). In the first approach, the addition of photothermal and magnetic forces is shown in real time by the structure color red-shifting. In the latter case, an initial magnetically induced blue shift was gradually overcompensated by increasing laser irradiation (λ = 808 nm) and vice versa. Therefore, we demonstrated that both actuations can be used independently or combined. Also, they can counteract each other or sum up the actuation strength by only changing the orientation of the magnetic field with respect to the structure. Notably, the Fe/PDMS cantilever presents a better responsivity to light actuation than other actuators of similar thickness.^13^ Unfortunately, comparing the magnetic actuation response to other is not possible, due to the lack of reported values that can be compared with our work.

**Figure 6:**
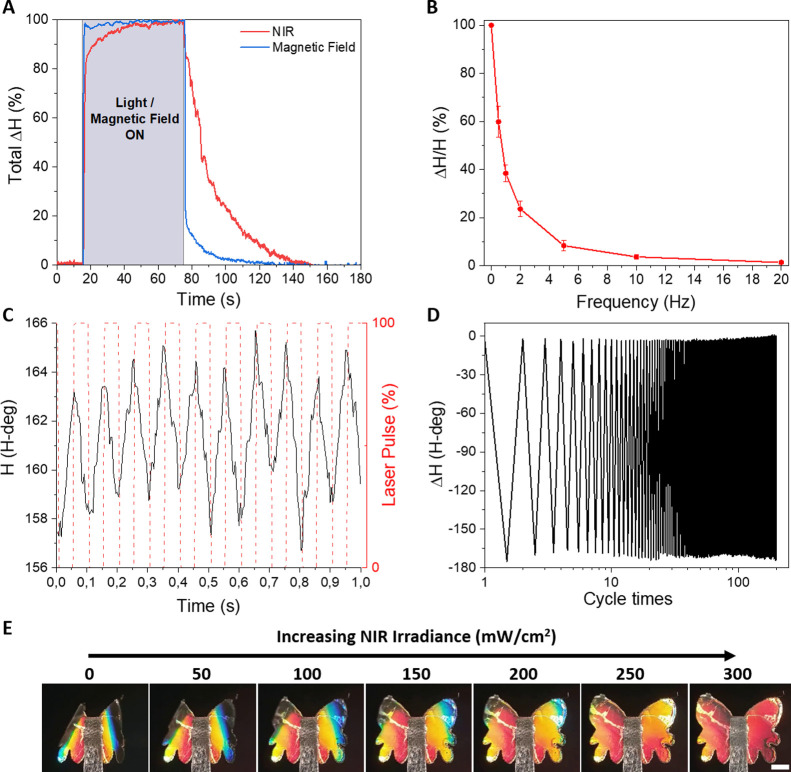
(A) Real-time detection of the color response (normalized) in a complete on–off cycle of NIR light (*I* = 277 mW·cm^–2^) and magnetic induction (*B* = 76 mT). (B) Colorimetric response induced by square-shaped laser radiation at increasing frequency. (C) Real-time colorimetric response to 10 Hz laser pulses (*I* = 277 mW·cm^–2^). (D) Long-term response under >200 on–off cycles (0.005 Hz; *I* = 277 mW·cm^–2^). (E) Butterfly-shaped nanostructured-Fe/PDMS metamaterial exhibiting colorimetric response upon increasing NIR intensity (from 0 to 300 mW/cm^2^ in steps of 50 mW/cm^2^; wavelength, 808 nm). Scale bar = 1 mm.

Other important factors to develop competitive externally controlled mechanical actuators are (i) their robustness and endurance, that is, their capacity to return to the initial state after actuation is repeated a number of times and (ii) the dynamic actuation/detection range under time-varying actuation sources. First, the dynamic response of the system during a single pulse of light and magnetic field is analyzed (Figure 6A). In both cases, the color abruptly changes during the initial second of actuation. However, while in the case of magnetic actuation the maximum deflection is almost instantaneous, in the case of light stimulation, the maximum deflection is only reached after 30 s due to the thermal swelling process. Similarly, the return to the original state is also much slower for the light stimulus due to the thermal cooling process of the material. In contrast, when the external magnetic field is removed, the cantilever rapidly returned to its initial position. The response capability of the actuator detection mechanism is also studied to time-varying actuation sources (in particular, to periodic light sources). The periodic stimulation of the system with a square-pulsed laser (intensity, 277 mW·cm^–2^) revealed a substantial decay of the maximum color change (i.e., maximum deflection) when increasing the frequency of the pulses due to the slow thermal process (Figure 6B). Despite the movement amplitude decay, the optical actuation and real-time mechanochromic detection can be performed up to 10 Hz (Figure 6C). In parallel, the endurance and robustness of the nanostructured-Fe/PDMS mechanochromic structure is confirmed by the reversible optical actuation at high light intensities as shown in Figure 6D for more than 200 cycles and as shown in Figure S6 for 10,000 cycles, which is equivalent to 27 h of continuous actuation. During the first 2 h of actuation, there is no significant change in the mechanochromic response. After 27 h of actuation, a minimal reduction (approximately 7%) of the colorimetric variation is observed.

Finally, we take advantage of the fabrication versatility of our concept to demonstrate the actuation/detection in more complex geometrical structures. Inspired by the structural coloration of the Morpho butterfly wing,^53^ we demonstrate the “flapping” of a “butterfly-shaped” mechanochromic system whose wing coloration changes upon external static (Figure 6E) or dynamic (Video S3) NIR stimulation.

## Conclusions

We have developed a mechanochromic soft Fe/PDMS actuator that is capable of responding to both light and magnetic stimuli, either individually or combined. We used a simple and low-cost fabrication process, easy to implement for large-scale fabrication, and highly versatile in the definition of the final shape design. The combination of a nanostructured-Fe layer mechanically linked to a PDMS layer enables merging the optical and magnetic actuations due to the unique combination of intense and broadband optical absorption of the bilayer and the high magnetic moment and tailored magnetic configuration of the nanostructured-Fe layer. Here, we can control the strength and actuation direction by modifying the direction of the magnetic field applied, due to the IP ferromagnetic vortex created by the Fe semi-shell nanostructures. The nanostructured-Fe layer, based in mechanically assembled Fe semi-shell nanostructures, also plays a very important role in the actuator mechanical response due to the reduction of Young’s modulus with respect to a continuous layer, however, keeping an efficient mechanical transmission of the bending moments (lost when not using coupled nanostructures) (Figure S4). Namely, when the Fe nanostructures are not coupled between them, the opto-mechanical bending of the cantilever (caused by the heat generated by the isolated Fe nanostructures along the PDMS cantilever thickness) is much weaker than when the Fe nanostructures are mechanically connected. In this case, the connected nanostructures behave like a continuous layer with an effective Young’s modulus, thereby amplifying the mechanical response by a pure bimetallic effect. Importantly, the actuators drastically change their intrinsic color in response to the induced mechanical deflection (mechanochromic response), by exploiting the diffraction of white light on its periodically nanostructured polymeric surface (grating), which is strongly angular-dependent. This is in contrast with the typical approaches used in smart mechanical sensing based in, for example, photonic crystals, where the mechanochromic response is generated by changing the crystal pitch or interplane distance.^54^ The strong mechanochromic response allows for a real-time colorimetric mapping of the actuation strength using a simple RGB (e.g., smartphone) camera. The Fe/PDMS actuator/detector not only shows high sensitivity using light (−0.62 H-deg·cm^2^/mW) or magnetic fields (−3.1 H-deg/mT), but also a remarkable endurance and versatility. The dual opto-magnetic responsivity of the system and its mechanochromic detection capabilities provide new appealing pillars for future developments of wireless multifunctional systems for soft robotic applications.

## Experimental Section

### Fabrication

First, the corrugated PDMS was obtained by spin-coating liquid PDMS (Sylgard 184) at 1000 rpm for 30 s on a silicone mold (with a pitch of 1600 nm), previously coated with (tridecafluoro-1,1,2,2-tetrahydrooctyl)trichlorosilane (97%, ABCR), and curing it at 80 °C for 30 min. The silicone mold was prepared from a commercial photonic grating (Thorlabs) that acts as a master.

In parallel, the nanostructured metal layer was fabricated by growing a Fe layer on a hexagonal close-packed structure of colloidal self-assembly of polystyrene nanospheres (diameter, 500 nm) (Life Technologies) deposited on a silicon substrate. To do the assembly, the close-packed monolayer of spheres is first formed at the air/water interface which is on top of an immersed silicon wafer. Once the monolayer is fully formed, the water is drained and the layer is deposited on the silicon substrate. After drying, the metal film (Fe) is deposited (80 nm) by electron beam physical vapor deposition (UNIVEX 450, Leybold).

The nanostructured metal film was then transferred to the flat side of the cured PDMS by contact transfer printing. A laser cutter (Epilog Mini 24, Epilog Laser) was used to define and cut the self-suspended structures into the desired geometry and dimensions. The structures were then released from the substrate by using a 0.5-mm-thick poly(methyl methacrylate) with double-sided pressure-sensitive adhesive as a base support.

### Morphological Characterization

The morphology of the nanostructured metal films and the PDMS photonic grating replica were examined by scanning electron microscopy [Quanta FEG 650, FEI (ThermoFisher)].

### Photothermal Characterization

The temperature monitoring of the sample was carried out using a non-contact infrared thermometer (MLX90614, Melexis) and a computer with LabVIEW data acquisition software. The infrared sensing characterization was carried out using a NIR laser diode with emission wavelength at 808 nm (B1-808-1500-15A, Sheaumann) and another laser with emission at 1470 nm (QSM-1470-3, QPhotonics). A germanium filter (WG90530-G, Thorlabs) was used to block the interferences at the infrared thermometer coming from the infrared light sources.

### Magnetic Characterization

The in-plane (IP) and out-of-plane (OOP) hysteresis loops were carried out using a Durham Magneto Optics Ltd. magneto-optic Kerr effect (MOKE) apparatus (NanoMOKE2).

### Colorimetric Measurements

The colorimetric measurements were done in transmission configuration, being the white light incidence angle 0° and the transmission angle 23° for all the measurements. The infrared lasers at 808 and 1470 nm were the same used for the photothermal characterization. The magnetic field in the magnetic actuation was generated using a spherical magnet of 12 mm (Supermagnete) clamped at the end of a movable bar that allows the movement of the magnet with respect to the cantilever.

The image capture was done using a conventional USB camera (Dino-Lite) and a smartphone camera. The image analysis was performed with colorevo [https://gitlab.com/c-p/colorevo], a purpose-built software for monitoring the evolution of the average hue in one or more predefined ROI of the captured video. This software is written in Python and its source code is freely available under the General Public License [https://www.gnu.org/licenses/gpl.html].

Rectangular ROIs of approximately 10 × 40 pixels were defined near the tip of the cantilever, where the color variation is larger. The ROI covered the whole width of the cantilever but is limited along the cantilever length to select a narrow color band, while still consisting of enough pixels to adequately cancel out the noise contribution from the camera CCD sensor and electronics.

The minimum detectable color change was determined as 3·*N*, where *N* is the noise in the hue (*H*) measurements taken during 20 s (i.e., 0.35 H-deg). The calculation of the limit of detection in the light-induced angular deflection θ changes was then obtained by
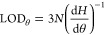
1

Analogously, the detection limit in light-induced temperature *T* variations was calculated by
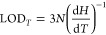
2

The slopes of *H*(*T*) and *H*(θ) are extracted from Figure S3A,B, respectively.

## Supporting Information Available

Equation of the radius of curvature for bimorph cantilevers, equations for the magnetic actuation, open source software for color analysis in ROI, spectral absorption of the nanostructured Fe/PDMS material, color changes upon illuminating and its correlation to the temperature, and comparative induced bending between coupled and non-coupled Fe nanostructures (PDF)
Opto-magnetic actuation with optical and magnetic forces adding to the cantilever movement (MP4)
Opto-magnetic actuation, where optical actuation and magnetic forces counteract each other, reducing the cantilever movement (MP4)
“Flapping” of a “butterfly-shaped” mechanochromic system (MP4)
