# Supplementary material for: Mechanochromic Detection for Soft Opto-Magnetic Actuators
Source: ACS Appl Mater Interfaces. 2021 Oct 1;13(40):47871–81. doi: 10.1021/acsami.1c11710 (PMC8517958; doi:10.1021/acsami.1c11710)
Supplement: Supplementary file 1 — am1c11710_si_001.pdf [file am1c11710_si_001.pdf]

Supporting Information

Mechanochromic detection for soft opto-magnetic actuators

Pau Güell-Grau, Pedro Escudero, Filippos Giannis Perdikos, José Francisco López-Barbera, Carlos Pascual-Izarra, Rosa Villa, Josep Nogués, Borja Sepúlveda, Mar Alvarez**

*E-mail: mar.alvarez@imb-cnm.csic.es (M. Alvarez), borja.sepulveda@icn2.cat (B. Sepúlveda)

Equation of the radius of curvature for bimorph cantilevers.

When bimorph cantilevers are heated, the difference in the thermal expansion coefficients (α) generate a differential stress, inducing a cantilever deflection. For a bi-material system, the temperature induced bending can be estimated using its radius of curvature, *r*, which depends on the stress from the temperature change (ΔT) and the residual stress that may be present in the material layers. It can be estimated by:

$$r = \frac{[(E_1 t_1{}^2)^2 + (E_2 t_2{}^2)^2 + 2E_1E_2t_1t_2(2t_1{}^2 + 3t_1t_2 + 2t_2{}^2)]}{[6E_1E_2t_1t_2(t_1 + t_2)(\alpha_1 - \alpha_2)\Delta T]} \quad (1)$$

where t_i (I = 1 and 2) is the layer thickness of each layer (1 = PDMS and 2 = nanostructured-Fe), and E_i is the Young's modulus of the ith layer. As the thickness of the nanostructured-Fe layer is much smaller than that of PDMS, $t_1 \gg t_2$, equation (1) can be simplified to

$$r = \frac{\frac{E_1 t_1{}^2}{6E_2 t_2} + \frac{2t_1}{3}}{(\alpha_1 - \alpha_2)\Delta T} \quad (2)$$

Thus, r decreases as t_1 and E_1 decrease. Furthermore, r decreases as the difference in the thermal expansion coefficients and temperature change (ΔT) increase.

Equations for the magnetic actuation.

A magnetic material of volume V experiences a magnetic torque under an external magnetic field due to the misalignment of the magnetization of the material ($\vec{\boldsymbol{M}}$) in respect to the applied magnetic field ($\vec{\boldsymbol{H}}$), which tends to the alignment of both vectors. It can be expressed as:

$$\vec{\boldsymbol{\tau}}_{\boldsymbol{mag}} = V\vec{\boldsymbol{M}} \times \vec{\boldsymbol{H}}$$

The magnetic torque ($\vec{\boldsymbol{\tau}}_{\boldsymbol{mag}}$) is orthogonal to both vectors, and it is maximized when $\vec{\boldsymbol{M}}$ and $\vec{\boldsymbol{H}}$ are perpendicular to each other.

In addition, in the presence of a magnetic field gradient, magnetic materials experience a force that pulls the material upward the magnetic field gradient, which is proportional to the magnetization and the magnetic field strength:

$$\vec{\boldsymbol{F}}_{\boldsymbol{mag}} = \mu_0 \nabla(\overrightarrow{\vec{\boldsymbol{M}} \cdot \boldsymbol{H}})$$

In this case, the force is maximized when the magnetic moment is parallel to the external magnetic field.

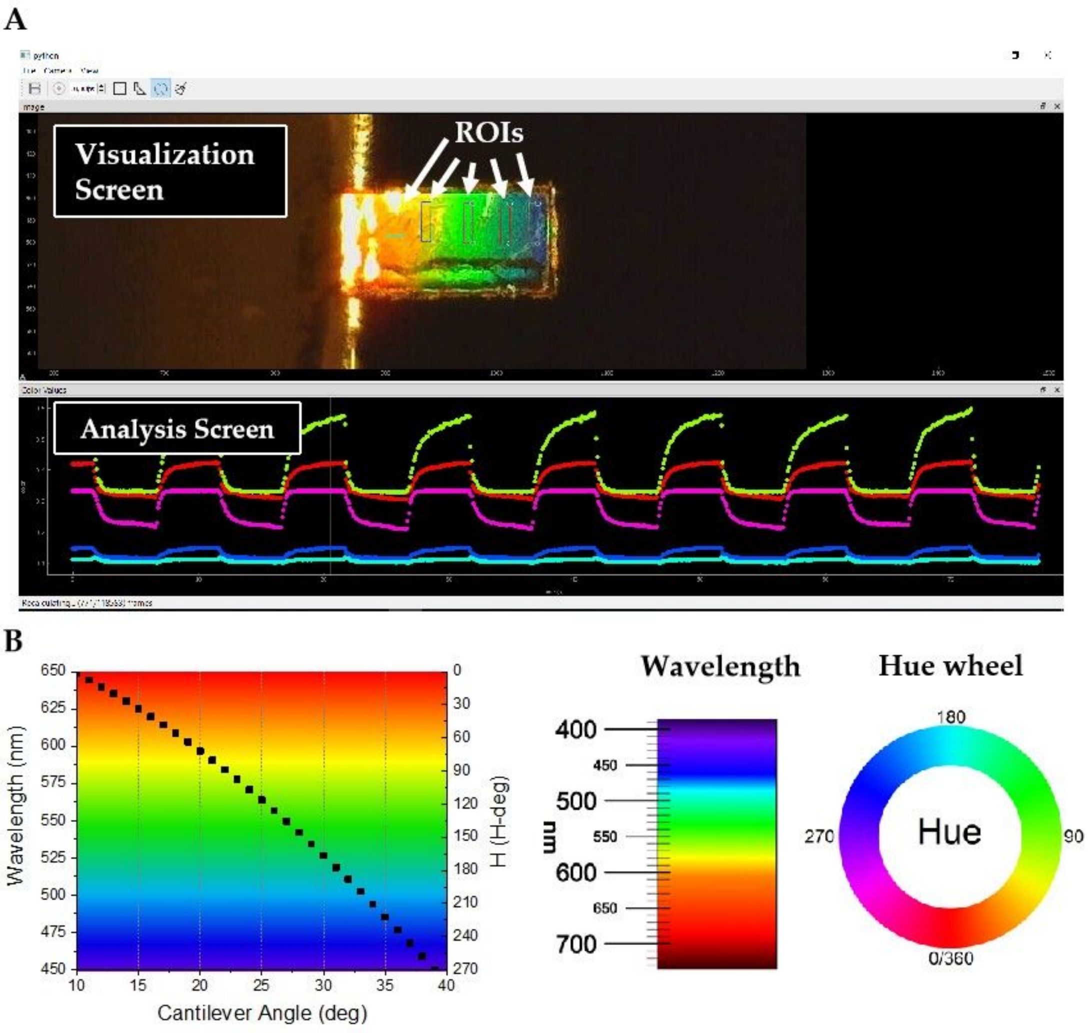

Figure S1. A) Open source software for color analysis in regions of interest (ROI) of a video source. B) Relation between the wavelength and associated the Hue value.

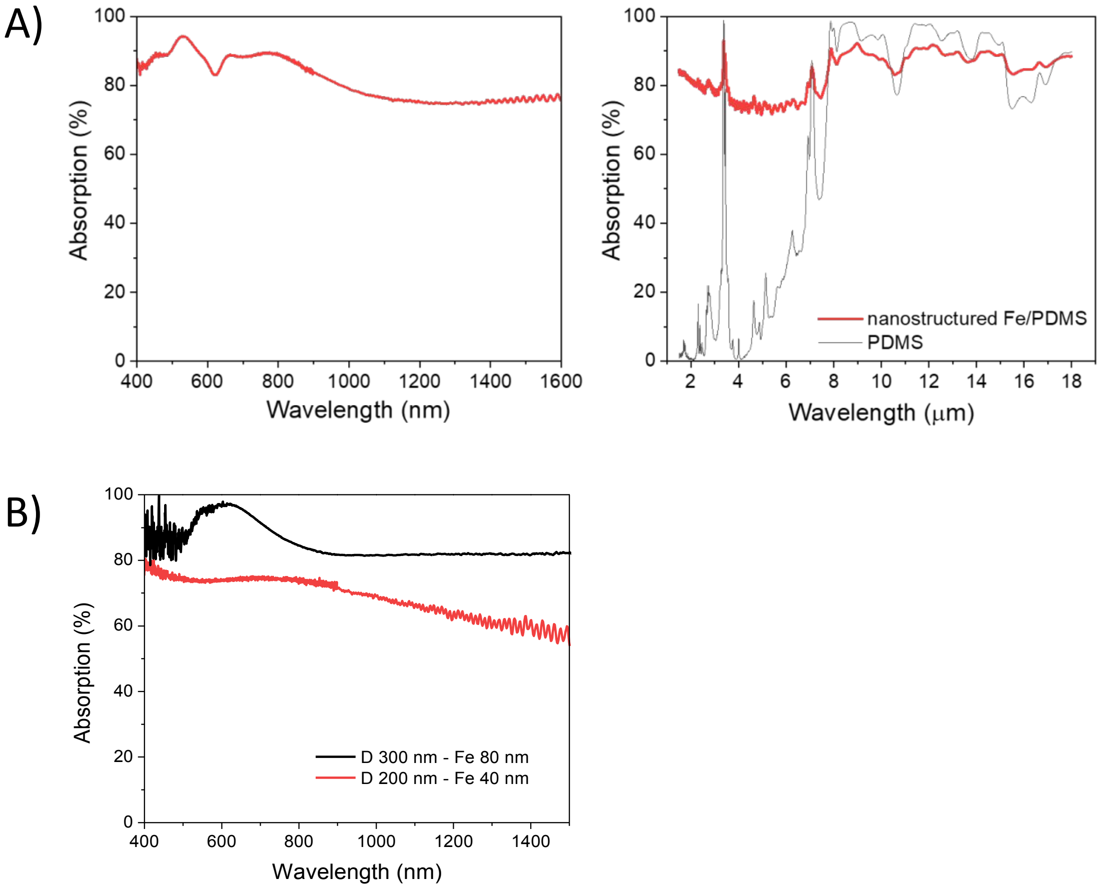

Figure S2. A) Spectral absorption of the nanostructured Fe/PDMS material with polystyrene spheres diameter of 500 nm and Fe thickness of 80 nm. B) Comparison of the absorption spectra for the nanostructured Fe/PDMS with the polystyrene spheres diameter is 300 nm and 200 nm and the Fe thickness is 80 nm and 40 nm, respectively.

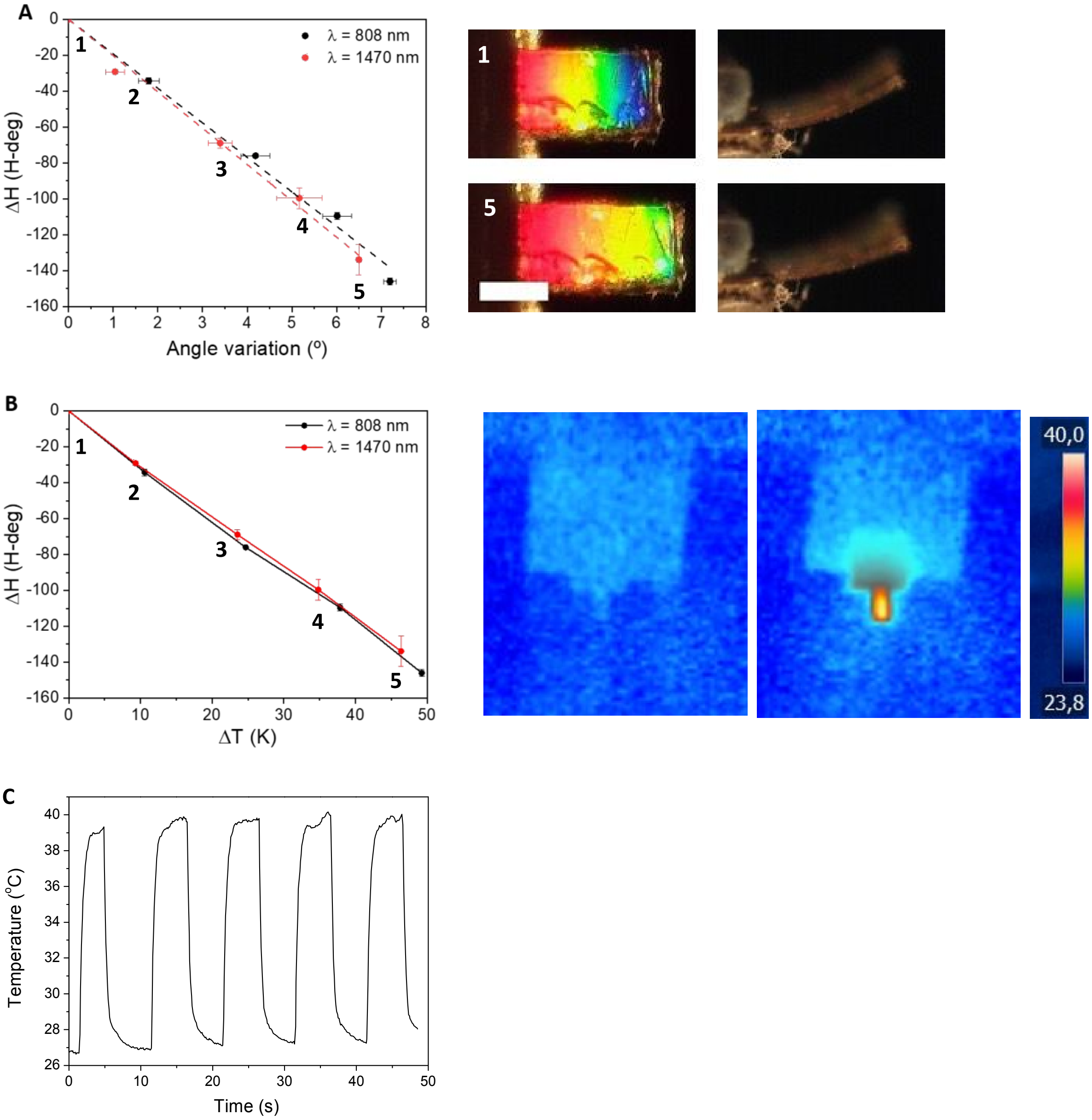

Figure S3. A) Experimentally measured color changes when illuminating the actuator with two sources of different wavelength at increasing light intensities: (**1**) 0, (**2**) 47.4, (**3**) 119.5, (**4**) 176.8, and (**5**) 235.4 mW/cm^2. The pictures on the right represent the cantilevers (top color view and lateral angle view in the absence of light actuation (1) and for a 235.4 mW/cm^2 illumination using the 1470 nm laser. B) Correlation between the light generated temperature changes and the induced color changes produced by the curvature variation. On the right, the thermal pictures in the absence of illumination and with 82.2 mW/cm^2 light intensity are shown. C) Dynamic thermal change generated by a 808 nm pulsed laser (frequency 0.1 Hz, intensity 70.3 mW/cm^2) acquired with a Thermal FLIR camera from a region of interest located at the tip of the cantilever.

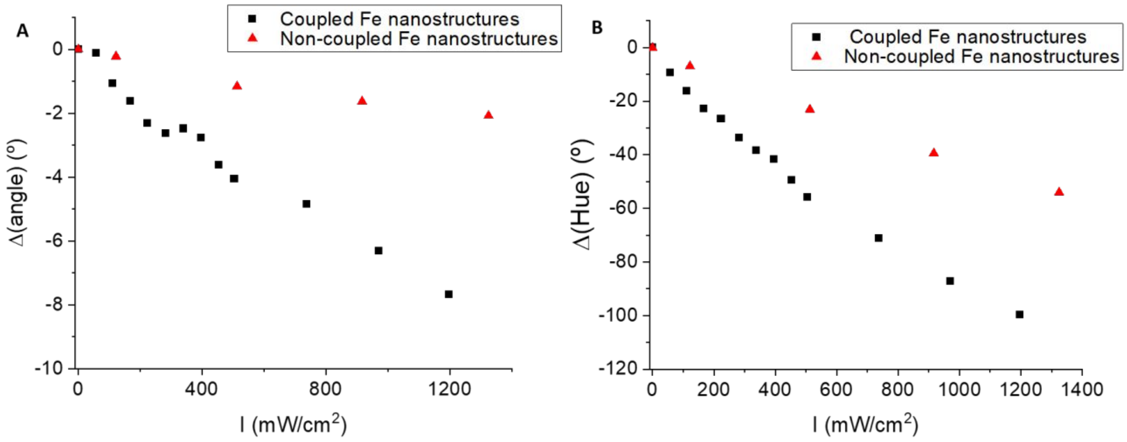

Figure S4. A) Experimentally measured bending angle and, B) color change of a PDMS cantilever with a layer of coupled Fe nanostructures (squares) and non-coupled Fe nanostructures (triangles).

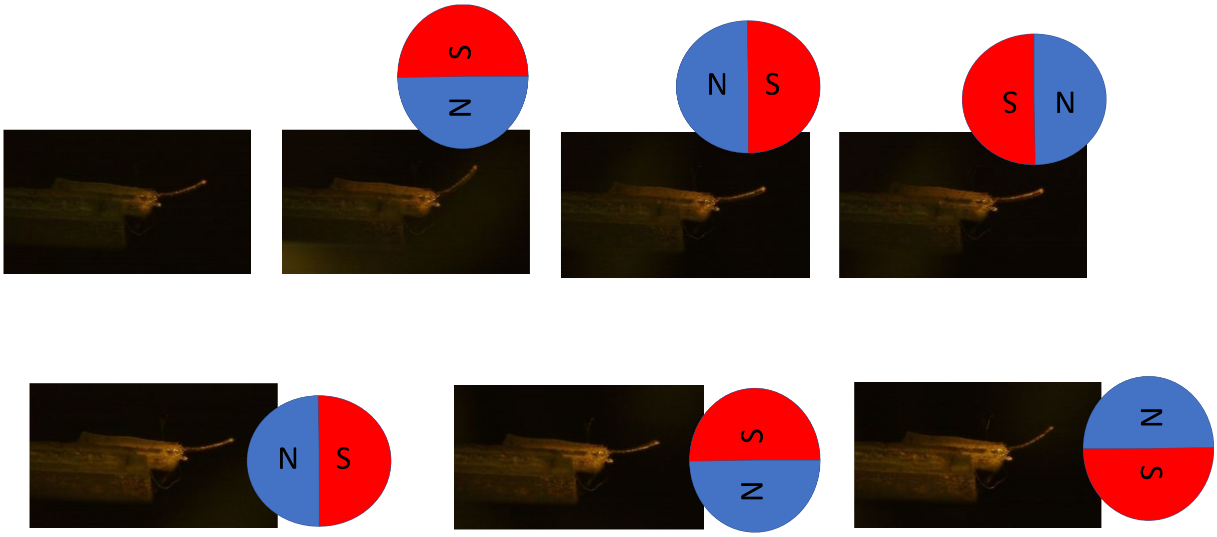

Figure S5. Representative lateral view images showing the magnetic bending of the cantilever with respect to the direction and orientation of the magnetic field generated by the 12 mm diameter FeNdB magnet.

<u>Explanation of the magnetic actuation for different directions and orientation of the magnetic field with respect to the cantilever</u>:

“For example, for the case where the magnet moves along the cantilever (Fig. 4C):

(i)

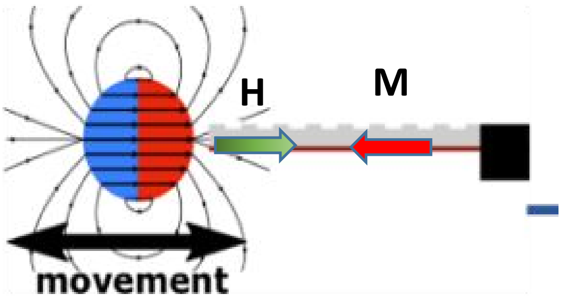

In this case H and M are parallel to each other, thus the cross-product results in H×M = 0. Consequently, in this case we expect **<u>no</u>** torque. Moreover, the field gradient is in the same direction as M and since the cantilever is clamped on one side, no bending of the cantilever

should take place. Hence, in first approximation, the response of the cantilever to the magnetic field should be negligible, as observed experimentally (Fig. 4C). Note that the weak response observed experimentally is probably due to the fact that the cantilevers are slightly bent in the as made state.

(ii)

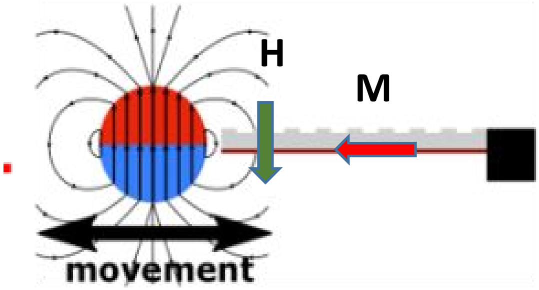

In this case M and H are perpendicular to each other. Consequently, the cross-product M×H is maximized, which leads to a strong response of the cantilever to the applied field, as observed experimentally. Namely, as the magnet gets closer, the cantilever feels a stronger magnetic field, consequently the torque will be larger. Moreover, in this case since M and H are at 90º, no effect of the field gradient is expected (since it is proportional to the dot product between M and H, M·H = 0). However, since the cantilever bends with increasing applied fields as a result of the torque, some effect of the field gradient is also expected, which should further strengthen the response of the cantilever.

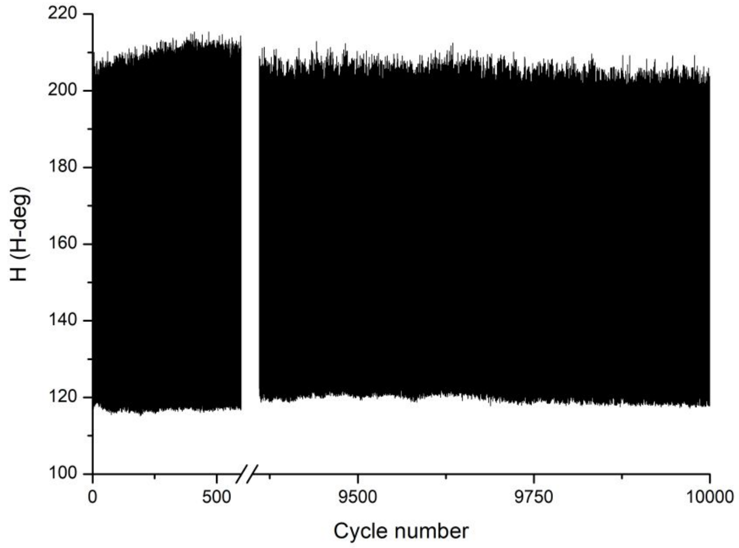

Figure S6. Plot of the long-term optical actuation with the 808 nm laser during 10000 cycles with an actuation frequency of 0.1 Hz and an intensity of 204 mW/cm^2.
